# VEGFR Inhibitors for Uterine Metastatic Perivascular Epithelioid Tumors (PEComa) Resistant to mTOR Inhibitors. A Case Report and Review of Literature

**DOI:** 10.3389/fonc.2021.641376

**Published:** 2021-03-26

**Authors:** Aikaterini Liapi, Patrice Mathevet, Fernanda G. Herrera, Delfyne Hastir, Apostolos Sarivalasis

**Affiliations:** ^1^Département d'oncologie, Centre Hospitalier Universitaire Vaudois (CHUV), Lausanne, Switzerland; ^2^Département de Gynécologie, Centre Hospitalier Universitaire Vaudois (CHUV), Lausanne, Switzerland; ^3^Institut Universitaire de Pathologie, Centre Hospitalier Universitaire Vaudois, Lausanne, Switzerland

**Keywords:** uterine perivascular epithelioid cell tumor, soft tissue sarcomas, mTOR, second line, VEGFR, PEComa

## Abstract

Uterine perivascular epithelioid cell tumors (PEComas) are rare neoplasms. PI3K/AKT/mTOR pathway upregulation is critical for their pathogenesis and is often associated with TSC1/TSC2 inactivation. Although first line mTOR inhibitors are an effective treatment, metastatic PEComas eventually progress. A 53-year-old woman presented a 4-month history of post-menopausal vaginal bleeding. Clinical and radiological examination detected a uterine mass and a single S1 bone lesion. The patient underwent a radical hysterectomy and bone biopsy. The anatomopathological evaluation concluded to an oligo-metastatic uterine PEComa. The tumor harbored a heterozygous deletion of 9q34 that contains the TSC1 gene. Concerning the primary lesion, the resection was complete and the single bone metastasis was treated with radiotherapy. Three months later, the patient presented bone, lung and subcutaneous metastatic progression. An everolimus and denosumab treatment was initiated. After 2 years of treatment, a clinically significant bone, lung and subcutaneous progression was detected. Following a literature review of the possible therapeutic options, we initiated a second line treatment by pazopanib. This treatment resulted in regression of the subcutaneous lesions and stability of lung and bone metastases. In this challenging, rare setting, our report suggests single agent, anti-angiogenic, tyrosine kinase inhibitor to be effective as second line treatment of metastatic uterine PEComa progressing on mTOR inhibitors.

## Introduction

Perivascular epithelioid cell tumors (PEComas) are rare mesenchymal tumors, originating from the perivascular epithelioid cell-line (PEC) (1, 2). PEComas are characterized by co-expression of melanocytic and myoid markers including actin, desmin, HMB-45, and Melan-A (3). S100 and CK are rarely expressed (4). The World Health Organization (WHO) defines PEComas as “mesenchymal tumors composed of histologically and immunohistochemically distinctive perivascular cells.” PEComas is a relatively new subgroup of tumors, first described in 1992. Under the initial description, PEComas only included angiomyolipoma (AML) and clear cell “sugar” tumor of the lung (CCST). Currently, lymphangioleiomyomatosis (LAM) has also been added to the PEComa family (4, 5). Although not all subsets of PEComas present malignant behavior, a considerable proportion exhibit malignant features including high proliferation rate and cytological atypia.

PEComas can occur at any age, but they are more common in the fourth decade. They affect more often women, which suggests a potential role of hormones in pathogenesis (6, 7). PEComas have tropism for the retroperitoneum, the kidneys and the genitourinary tract, but rarely can affect other localizations. As far as uterine PEComas are concerned, they are usually located in the corpus. Rarely the uterine cervix can also be involved (1, 5). The differential diagnosis of uterine PEComas is large and includes all types of uterine malignant and benign tumors. Especially challenging is their radiological distinction from smooth muscle tumors namely leiomyomas, a frequent benign tumor present in up to 80% of middle age women (8). PEComas can also be misdiagnosed for carcinomas, especially if visceral or cervical involvement is present, mimicking clear cell or other subtypes radiological characteristics.

The pathogenesis of PEComas is not yet fully understood. It has been hypothesized that these tumors are associated with tuberous sclerosis complex (TSC). This tumor-suppressor gene syndrome is caused by inactivation of TSC1 and TSC2, encoding hamartin and tuberin, respectively (9). These two proteins form a heterodimeric complex with inhibitor effect to the mammalian target of rapamycin (mTOR) activity. Inactivation of the tuberin/hamartin complex, results from a germline and /or loss-of-function mutation of either TSC1 or TSC2 genes. Subsequent permanent activation of mTOR promotes cell growth. Similar mutations have also been detected in sporadic, not associated with TSC, PEComas (10, 11).

Many different angiogenesis inhibitors were reported effective for advanced PEComa treatment (4). Angiogenesis is a complex web of cytokines and receptors with intracellular and nuclear cascades for signal transmission. They are intimately related to tumor genesis, proliferation and invasion. Vascular endothelial growth factor (VEGF) over-expression is a prognostic factor, associated with increased risk of metastases and decreased overall survival, in patients with solid tumors. The VEGF receptors (VEGFR) are a family of tyrosine kinase (TK) receptors that include VEGFR-1, VEGFR-2, and VEGFR-3 expressed among tumor microenvironment stromal cells and endothelial cells. The platelet derived growth factor (PDGF) and its receptor (PDGFR) family includes PDGFR, c-Kit, and Flt-3. Their main effect is the proliferation and migration of cells.

The treatment of PEComas can be challenging, as there are no prospective trials nor established standard treatment guidance. Despite mTOR inhibitors, resistant or progressive diseases treatment is unclear. In this challenging context, where commonly used chemotherapies including anthracyclines, taxanes, and anti-metabolites have marginal or no effect, prospective research is urgently needed to determine second line treatment options (12). In our report, we present the efficacy of the 2nd line treatment by pazopanib, a multi-tyrosine kinase inhibitor, in a patient with a metastatic uterine PEComa, progressing after 2 years of everolimus treatment.

## Case Report

A 53 year-old woman presented a 4-month history of vaginal bleeding without associated abdominal pain or other symptoms. On the gynecological examination, a right side, para-uterine mass was detected. The vaginal ultrasound and MRI showed the 6 × 6 cm mass with no associated lymph node involvement. The PET-CT identified a single bone lesion in the sacral spine (S1). Tumor markers including CEA, CA125, CA19-9, and CA15-3 were within normal range.

**Figure 1:**
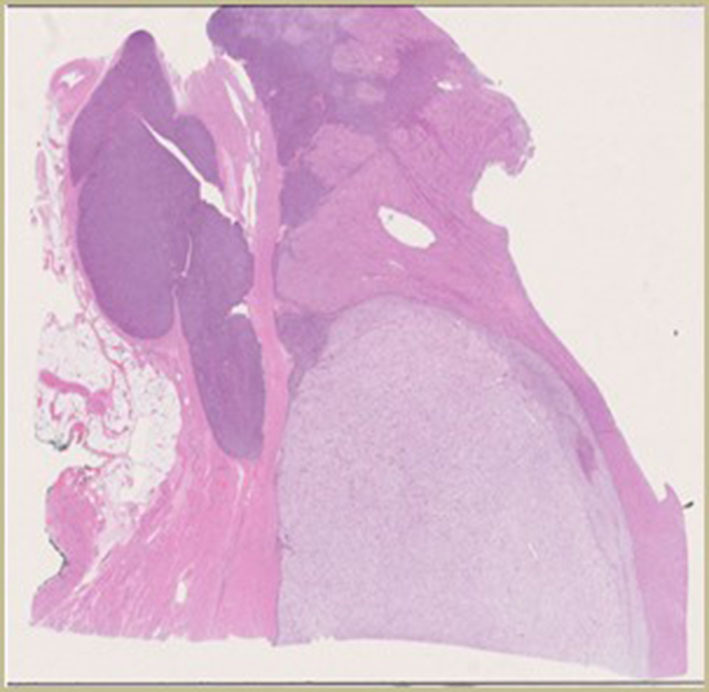
Initial pathology.

The patient underwent radical hysterectomy and bone biopsy. Both bone biopsy and hysterectomy indicated an oligo-metastatic uterine PEComa, without cervical involvement, with high-grade nuclear features and lymphovascular invasions (Figure 1). The tumor expressed CD10, the myoid smooth muscle actin and HMB45. Focal anti-TFE3 was also observed (11, 13). The single bone metastasis was treated with radiotherapy (5 fractions of 7 Gy for a total of 35 Gy).

**Figure 2:**
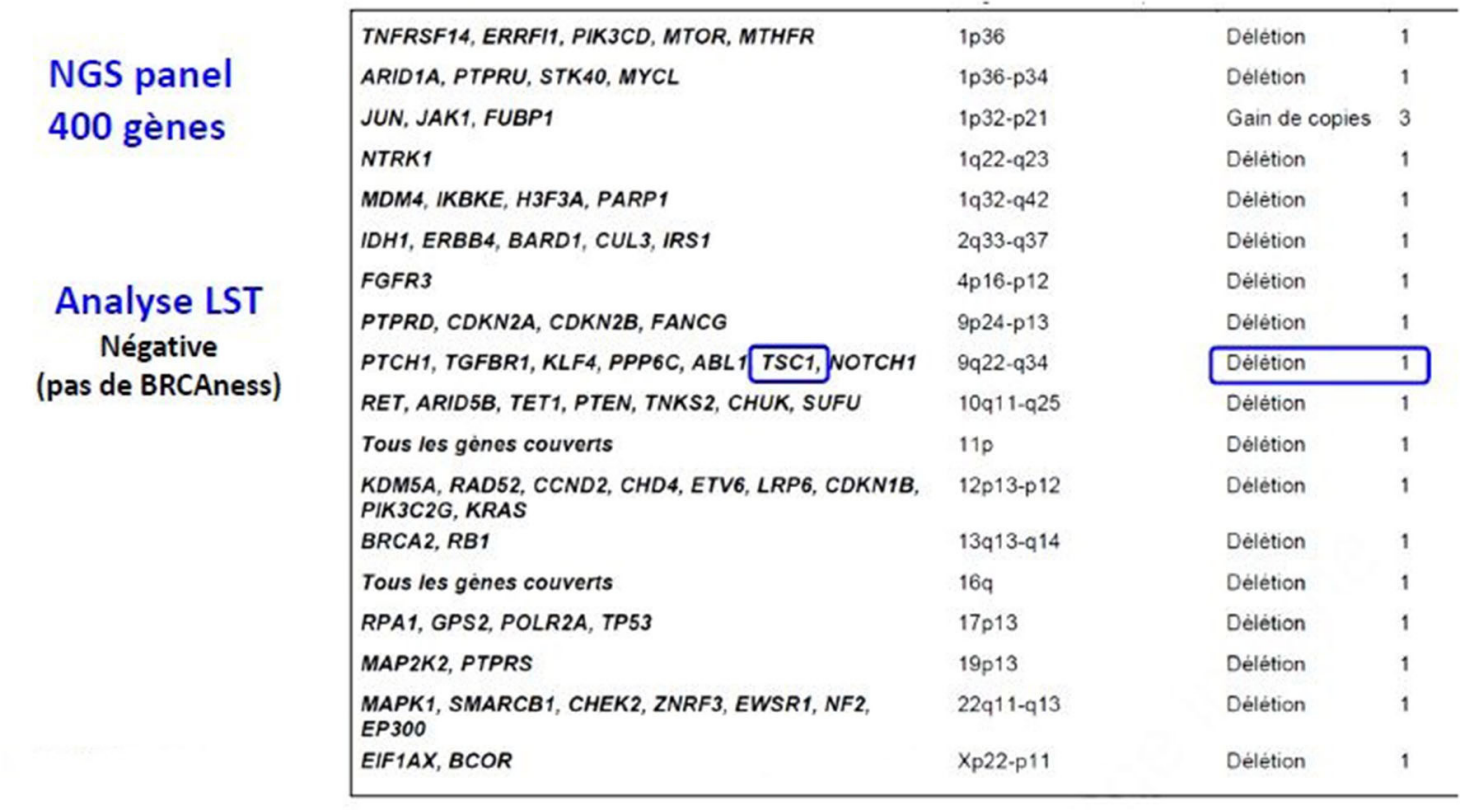
NGS 400 genes.

Three months later, the patient presented asymptomatic bone, lung and subcutaneous recurrence. Targeted NGS (52-cancer gene hotspot panel) detected no mutation but a heterozygous deletion of 9q34 that contains the TSC1 gene (Figure 2) (14), prompting a first line everolimus treatment (10 mg 1×/day, po) (15). For the bone metastasis, denosumab (120 mg 1/month sc) was initiated, and a quarterly follow-up by PET-CT as well as regular clinical surveillance was decided. On this treatment, the disease was controlled for 2 years, but unfortunately the patient started feeling pain in the subcutaneous lesion of the right thigh and clinical progression was noted. The PET-CT showed bone, lung and subcutaneous progression. A second line treatment by pazopanib and denosumab was introduced, based on reports on anti-angiogenic tyrosine kinase inhibitors (TKIs) effect in PEComas (16, 17) (Table 1).

**Table 1:** Summary of studies evaluating anti-VEGFR treatments for PEComas/soft tissue sarcomas (18–24).

**Trail**	**Anti-VEGFR**	**Phase**	**Total No. of patients**	**No. of patients with PEComa**	**No. of patients receiving anti-VEGFR**	**Line of treatment**	**Response**
Pazopanib for metastatic soft-tissue sarcoma (PALETTE): a randomized, double-blind, placebo-controlled phase 3 trial	Pazopanib	III	372	Not specified	246	Any	14 PR, 164 SD, 57 PD. Median PFS 4.6 mos, OS 12.5 mos
Treatment patterns and clinical outcomes with pazopanib in patients with advanced soft tissue sarcomas in a compassionate use setting: results of the SPIRE study	Pazopanib	II	211	2	211	Mostly second- and third-line	0 CR, 15 PR, 38 SD. Median OS 11.1 mos,
Role of chemotherapy, VEGFR inhibitors, and mTOR inhibitors in advanced perivascular epithelioid cell tumors (PEComas)	Pazopanib, sorafenib, sunitinib	Observational	53	53	12: 9 pazopanib, 2 sorafenib, 1 sunitinib	3 second-line, 4 third-line, and 5 fourth- or more lines.	1 PR (sorafenib), 9 SD, 2 PD. Prolonged disease stabilization (12 mos): patients (pazopanib, sorafenib)
Combination targeted therapy of VEGFR inhibitor, sorafenib, with an mTOR inhibitor, sirolimus induced a remakable response of rapid progressive Uterine PEComa	Sorafenib	Case-report	1	1	1	First	CR 7 months (then lost of follow up)
1450P—systemic therapy in perivascular epithelioid cell tumors (PEComa)	Pazopanib, sunitinib	Observational	49	49	4: pazopanib or sunitinib	1–7 (no more info)	1 PR and 2 SD, with a median PFS 7.3 mos
Metastatic perivascular epithelioid cell tumor of the kidney: a case report with emphasis on response to the tyrosine-kinase inhibitor sunitinib	Sunitinib	Case-report	1	1	1	First	SD 9 months
Anthracycline, gemcitabine, and pazopanib in epithelioid sarcoma	Pazopanib	Observational	115	Not specified	18	Any	PFS 3 months, OS 14 months
Phase II trial of VEGFR2 inhibitor apatinib for metastatic sarcoma: focus on efficacy and safety	Apatinib	II	64	not specified	64	After chemotherapy failure	0 CR, 10 PR, 41 SD, 8 PD, median PFS 7.93 mos
Real-world experiences with pazopanib in patients with advanced soft tissue and bone sarcoma in Northern California	Pazopanib	Observational	123	1	123	Any (median number of prior lines: three)	1 CR, 12 PR, 34 SD, 76 PD. Median PFS 3 months
Pazopanib, a multikinase angiogenesis inhibitor, in patients with relapsed or refractory advanced soft tissue sarcoma: a phase II study from the European Organization for research and treatment of cancer–soft tissue and bone sarcoma group (EORTC Study 62043)	Pazopanib	PHASE II	142	Not specified	142	No more than 2 prior lines of cytotoxic treatment	0 CR 9 PR
The clinical outcome of pazopanib treatment in Japanese patients with relapsed soft tissue sarcoma: A Japanese Musculoskeletal Oncology Group (JMOG) study	Pazopanib	Retrospective	156	Not specified	156	Any	13 PR, 74 SD, median PFS 15.4 weeks

**Figure 3:**
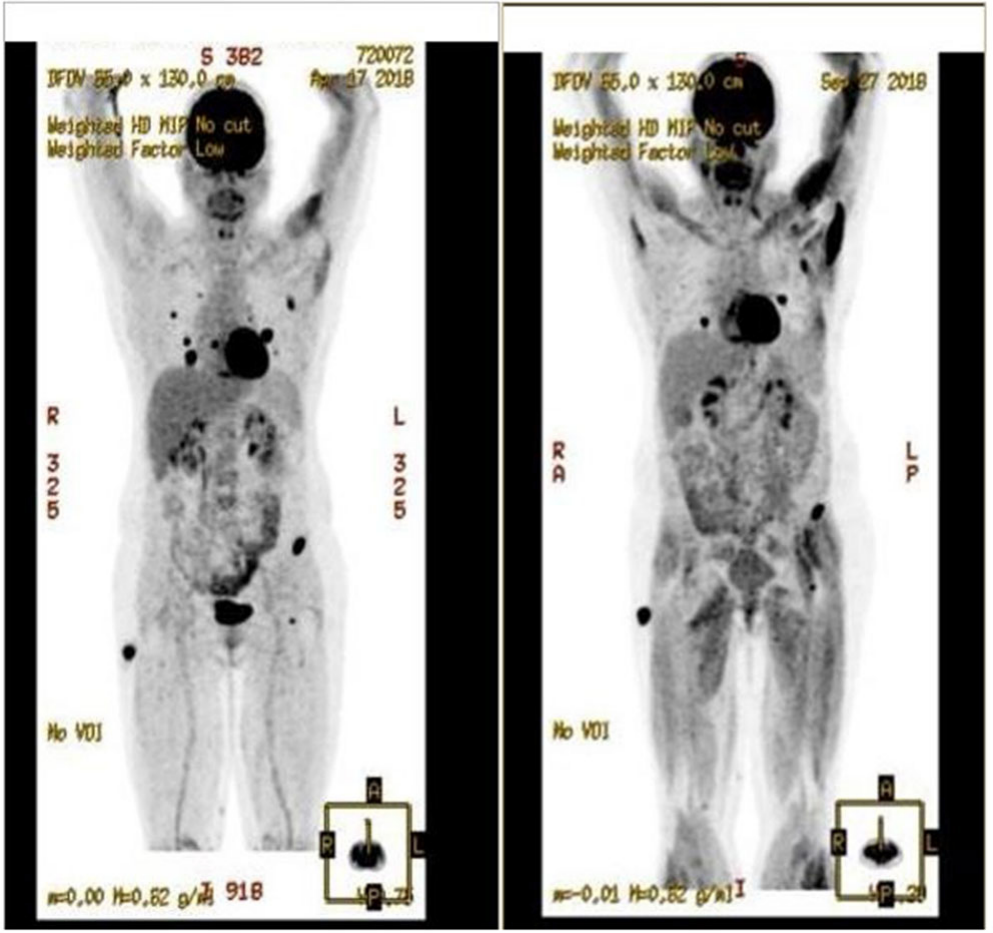
Response on pazopanib treatment.

During the treatment, the patient had regular clinical, biological and radiological controls. With the combination of pazopanib (800 mg 1×/day po) and denosumab, there was regression of the subcutaneous disease and stability of lung and bone metastasis for over a year Figure 3. It is important to note that all the treatments were well-tolerated, only with mild toxicities. No dose adaptation was necessary.

## Discussion

Both sporadic and TSC associated PEComas present mTOR pathway alterations. This provided the rational for rather successful use of mTOR inhibitors including sirolimus, everolimus or temsirolimus. Despite being a rare tumor with no specific randomized controlled trial assessing the optimal treatment sequence, mTOR inhibitors are currently considered the most effective treatment option for metastatic PEComas (25–29). In the case of our patient, single agent everolimus treatment at the standard dose of 10 mg per day was effective for up to 2 years Albeit this observation, it should be noted that in the reported cases the response rate and PFS varies, with a PFS of 9 months (4), according to the clinical context and previous prescribed treatments.

At the time of symptomatic progression, the therapeutic plan of our patient became even more challenging. Since no targetable alteration was detected in the 52-genes tumor DNA sequencing, we underwent a literature review on Scopus and PubMed. The key word used were “metastatic” and “PEComa,” “uterine perivascular epithelioid cell tumor,” “soft tissue sarcomas,” “mTOR,” “treatment.” We selected publications from 2000 to 2019.

The review pointed out potential benefit from anti-angiogenic treatment as an effective therapeutic option for our patient (16, 17). Furthermore, the local funding system enables pazopanib treatment for soft tissue sarcomas.

Pazopanib is an oral, multi-targeted tyrosine kinase inhibitor, which has activity against VEGF-1–3, PDGFRα- β and KIT, resulting in tumor growth blockage and angiogenesis inhibition. The Phase III, placebo-controlled, double-blind PALETTE trial, comparing the efficacy of pazopanib vs. placebo in patients with non-adipocytic soft tissue sarcoma already treated with doxorubicin, showed a significant increase in PFS with pazopanib (4.6 vs. 1.6 months; *P* < 0.001); ORR was 6%. Median OS was 12.5 months with pazopanib vs. 10.7 months with placebo, but was not statistically significant (HR: 0.86, 95% CI: 0.67–1.11; *p* = 0.25). There is no precision on whether patients with PEComas were included in the trial (16). The SPIRE trial showed the activity of pazopanib in patients with soft tissue sarcomas, based on reports on specific histological subtypes (17). Two of the patients included in the trial were diagnosed with PEComas.

Sunitinib and regorafenib are approved for gastrointestinal stromal tumor (GIST), but not for other STS, as the clinical trials of these agents as STS treatment are limited to single-arm phase II trials (30, 31).

Sorafenib is another multi-TKI that has only showed limited activity in a phase II trial (32) including patients with six different types of STS. There was no precision on whether patients with PEComas were included.

In our review three interesting therapeutic combinations were included. One case-report reported the combination of sorafenib with sirolimus as effective palliative therapy in malignant PEComa (33). In another report bevacizumab, a monoclonal antibody (MoAb) targeting VEGF was evaluated. The combination of bevacizumab with doxorubicin was evaluated in a phase II trial, among 17 patients with metastatic, anthracycline-naïve, soft tissue sarcomas (34). The overall response rate was 12%, equal to the observed for single-agent doxorubicin arm. However, stable disease, lasting four cycles or longer, was observed in 65% of patients. Interestingly, in a retrospective case series published in 2018, seven patients with advanced PEComa progressing on mTOR inhibitor (sirolimus) were treated with exemestane, an aromatase inhibitor, in combination with everolimus. Authors reported restoration of tumors to mTOR inhibitor leading to a median PFS of 7 months and median duration of the response 11 months (35).

Although with limited impact and no comparison arm, our treatment decision lead to a 12 months stabilization of our patient lung and bone disease and notably with regression of her symptomatic subcutaneous metastasis.

## Conclusion

PEComas are rare mesenchymal neoplasms, for which there is no systemic treatment established. Our report provides data that mTOR inhibitors and anti-angiogenic TKI can be effective treatments, with an acceptable toxicity profile. Further research is needed in order establish the optimal treatment sequence in this setting.

## Author Contributions

AL elaborated, drafted, reviewed the manuscript, and analyzed the patient's data. PM and FH drafted and reviewed the manuscript. DH elaborated and analyzed the pathology. AS elaborated, drafted, reviewed the manuscript, and coordinated publication. All authors contributed to the article and approved the submitted version.

## Conflict of Interest

The authors declare that the research was conducted in the absence of any commercial or financial relationships that could be construed as a potential conflict of interest.

## References

[1] Conlon N Soslow RA Murali R. Perivascular epithelioid tumours (PEComas) of the gynaecological tract. J Clin Pathol. (2015) 68:418–26. 10.1136/jclinpath-2015-202945 25750268 PMC4984252

[2] Bonetti F Pea M Martignoni G Zamboni G. PEC and sugar. Am J Surg Pathol. (1992) 16:307–8. 10.1097/00000478-199203000-00013 1599021

[3] Hornick JL Fletcher CD. PEComa: what do we know so far? Histopathology. (2006) 48:75–82. 10.1111/j.1365-2559.2005.02316.x 16359539

[4] Folpe AL Mentzel T Lehr HA Fisher C Balzer BL Weiss SW. Perivascular epithelioid cell neoplasms of soft tissue and gynecologic origin: a clinicopathologic study of 26 cases and review of the literature. Am J Surg Pathol. (2005) 29:1558–75. 10.1097/01.pas.0000173232.22117.37 16327428

[5] Sanfilippo R Jones RL Blay JY Le Cesne A Provenzano S Antoniou G. Role of chemotherapy, VEGFR inhibitors, and mTOR inhibitors in advanced perivascular epithelioid cell tumors (PEComas). Clin Cancer Res. (2019) 25:5295–300. 10.1158/1078-0432.CCR-19-0288 31217199

[6] Baird DD Dunson DB Hill MC Cousins D Schectman JM. High cumulative incidence of uterine leiomyoma in black and white women: ultrasound evidence. Am J Obstet Gynecol. (2003) 188:100–7. 10.1067/mob.2003.99 12548202

[7] Sadigh S Shah P Weber K Sebro R Zhang PJ. Primary malignant perivascular epithelioid cell neoplasm (PEComa) of the bone mimicking granular cell tumor in core biopsy: a case report and literature review. Oncol Lett. (2018) 15:2946–52. 10.3892/ol.2017.7662 29435023 PMC5778776

[8] Pan CC Chung MY Ng KF Liu CY Wang JS Chai CY. Constant allelic alteration on chromosome 16p (TSC2 gene) in perivascular epithelioid cell tumour (PEComa): genetic evidence for the relationship of PEComa with angiomyolipoma. J Pathol. (2008) 214:387–93. 10.1002/path.2289 18085521

[9] Musella A De Felice F Kyriacos Kyriacou A Barletta F Maria Di Matteo F Marchetti C. Perivascular epithelioid cell neoplasm (PEComa) of the uterus: a systematic review. Int J Surg. (2015) 19:1–5. 10.1016/j.ijsu.2015.05.002 25981307

[10] Bennett JA Braga AC Pinto A Van de Vijver K Cornejo K Pesci A. Uterine PEComas: a morphologic, immunohistochemical, and molecular analysis of 32 tumors. Am J Surg Pathol. (2018) 42:1370–83. 10.1097/PAS.0000000000001119 30001237 PMC6133752

[11] Schoolmeester JK Dao LN Sukov WR Wang L Park KJ Murali R. TFE3 translocation-associated perivascular epithelioid cell neoplasm (PEComa) of the gynecologic tract: morphology, immunophenotype, differential diagnosis. Am J Surg Pathol. (2015) 39:394–404. 10.1097/PAS.0000000000000349 25517951 PMC4982474

[12] Machado I Cruz J Lavernia J Rayon JM Poveda A Llombart-Bosch A. Malignant PEComa with metastatic disease at diagnosis and resistance to several chemotherapy regimens and targeted therapy (m-TOR inhibitor). Int J Surg Pathol. (2017) 25:543–9. 10.1177/1066896917701245 28459168

[13] Nellist M Brouwer RW Kockx CE van Veghel-Plandsoen M Withagen-Hermans C Prins-Bakker L. Targeted next generation sequencing reveals previously unidentified TSC1 and TSC2 mutations. BMC Med Genet. (2015) 16:10. 10.1186/s12881-015-0155-4 25927202 PMC4422413

[14] van Slegtenhorst M de Hoogt R Hermans C Nellist M Janssen B Verhoef S. Identification of the tuberous sclerosis gene TSC1 on chromosome 9q34. Science. (1997) 277:805–8. 10.1126/science.277.5327.805 9242607

[15] Starbuck KD Drake RD Budd GT Rose PG. Treatment of advanced malignant uterine perivascular epithelioid cell tumor with mTOR inhibitors: single-institution experience and review of the literature. Anticancer Res. (2016) 36:6161–4. 10.21873/anticanres.11208 27793946

[16] van der Graaf WT Blay JY Chawla SP Kim DW Bui-Nguyen B Casali PG. Pazopanib for metastatic soft-tissue sarcoma (PALETTE): a randomised, double-blind, placebo-controlled phase 3 trial. Lancet. (2012) 379:1879–86. 10.1016/S0140-6736(12)60651-5 22595799

[17] Gelderblom H Judson IR Benson C Merimsky O Grignani G Katz D. Treatment patterns and clinical outcomes with pazopanib in patients with advanced soft tissue sarcomas in a compassionate use setting: results of the SPIRE study. Acta Oncol. (2017) 56:1769–75. 10.1080/0284186X.2017.1332779 28723233

[18] Hindi N Sanfilippo R Stacchiotti S Fumagalli E Libertini M Provenzano S. 1450P - systemic therapy in perivascular epithelioid cell tumors (pecoma). Ann Oncol. (2014) 25:iv506. 10.1093/annonc/mdu354.39

[19] AlAzab RS Alorjani MS Sahawneh FE Al-Sukhun S. Metastatic perivascular epithelioid cell tumor of the kidney: a case report with emphasis on response to the tyrosine-kinase inhibitor sunitinib. Res Rep Urol. (2019) 11:311–7. 10.2147/RRU.S226005 32010646 PMC6859122

[20] Frezza AM Jones RL Lo Vullo S Asano N Lucibello F Ben-Ami E. Anthracycline, gemcitabine, and pazopanib in epithelioid sarcoma: a multi-institutional case series. JAMA Oncol. (2018) 4:e180219. 29800950 10.1001/jamaoncol.2018.0219 PMC6143006

[21] Liao Z Li F Zhang C Zhu L Shi Y Zhao G. Phase II trial of VEGFR2 inhibitor apatinib for metastatic sarcoma: focus on efficacy and safety. Exp Mol Med. (2019) 51:1–11. 10.1038/s12276-019-0221-7 30816108 PMC6395676

[22] Seto T Song MN Trieu M Yu J Sidhu M Liu CM. Real-world experiences with pazopanib in patients with advanced soft tissue and bone sarcoma in Northern California. Med Sci. (2019) 7:48. 10.3390/medsci7030048 30889920 PMC6473235

[23] Sleijfer S Ray-Coquard I Papai Z Le Cesne A Scurr M Schöffski P. Pazopanib, a multikinase angiogenesis inhibitor, in patients with relapsed or refractory advanced soft tissue sarcoma: a phase II study from the European organisation for research and treatment of cancer-soft tissue and bone sarcoma group (EORTC study 62043). J Clin Oncol. (2009) 27:3126–32. 10.1200/JCO.2008.21.3223 19451427

[24] Nakamura T Matsumine A Kawai A Araki N Goto T Yonemoto T. The clinical outcome of pazopanib treatment in Japanese patients with relapsed soft tissue sarcoma: a Japanese Musculoskeletal Oncology Group (JMOG) study. Cancer. (2016) 122:1408–16. 10.1002/cncr.29961 26970174 PMC5069581

[25] Kenerson H Folpe AL Takayama TK Yeung RS. Activation of the mTOR pathway in sporadic angiomyolipomas and other perivascular epithelioid cell neoplasms. Hum Pathol. (2007) 38:1361–71. 10.1016/j.humpath.2007.01.028 17521703 PMC2722219

[26] Wagner AJ Malinowska-Kolodziej I Morgan JA Qin W Fletcher CD Vena N. Clinical activity of mTOR inhibition with sirolimus in malignant perivascular epithelioid cell tumors: targeting the pathogenic activation of mTORC1 in tumors. J Clin Oncol. (2010) 28:835–40. 10.1200/JCO.2009.25.2981 20048174 PMC4810029

[27] Italiano A Delcambre C Hostein I Cazeau AL Marty M Avril A. Treatment with the mTOR inhibitor temsirolimus in patients with malignant PEComa. Ann Oncol. (2010) 21:1135–7. 10.1093/annonc/mdq044 20215136

[28] Chiu HF Wen MC Li JR Ho HC Shu KH. Successful treatment with sirolimus for an angiomyolipoma mimicking renal cell carcinoma in a transplanted kidney. Transpl Int. (2015) 28:1116–20. 10.1111/tri.12567 25790294

[29] Gennatas C Michalaki V Kairi PV Kondi-Paphiti A Voros D. Successful treatment with the mTOR inhibitor everolimus in a patient with perivascular epithelioid cell tumor. World J Surg Oncol. (2012) 10:181. 10.1186/1477-7819-10-181 22943457 PMC3499435

[30] Mahmood ST Agresta S Vigil CE Zhao X Han G D'Amato G. Phase II study of sunitinib malate, a multitargeted tyrosine kinase inhibitor in patients with relapsed or refractory soft tissue sarcomas. Focus on three prevalent histologies: leiomyosarcoma, liposarcoma and malignant fibrous histiocytoma. Int J Cancer. (2011) 129:1963–9. 10.1002/ijc.25843 21154746 PMC3776586

[31] Mir O Brodowicz T Italiano A Wallet J Blay JY Bertucci F. Safety and efficacy of regorafenib in patients with advanced soft tissue sarcoma (REGOSARC): a randomised, double-blind, placebo-controlled, phase 2 trial. Lancet Oncol. (2016) 17:1732–42. 10.1016/S1470-2045(16)30507-1 27751846

[32] Maki RG D'Adamo DR Keohan ML Saulle M Schuetze SM Undevia SD. Phase II study of sorafenib in patients with metastatic or recurrent sarcomas. J Clin Oncol. (2009) 27:3133–40. 10.1200/JCO.2008.20.4495 19451436 PMC2716936

[33] Gao F Huang C Zhang Y Sun R Zhang Y Wang H. Combination targeted therapy of VEGFR inhibitor, sorafenib, with an mTOR inhibitor, sirolimus induced a remakable response of rapid progressive uterine PEComa. Cancer Biol Ther. (2016) 17:595–8. 10.1080/15384047.2016.1167290 27030639 PMC4990405

[34] D'Adamo DR Anderson SE Albritton K Yamada J Riedel E Scheu K. Phase II study of doxorubicin and bevacizumab for patients with metastatic soft-tissue sarcomas. J Clin Oncol. (2005) 23:7135–42. 10.1200/JCO.2005.16.139 16192597

[35] Sanfilippo R Fabbroni C Fucà G Fumagalli E Morosi C Sbaraglia M. Addition of antiestrogen treatment in patients with malignant PEComa PROGRESSING to mTOR inhibitors. Clin Cancer Res. (2020) 26:5534–8. 10.1158/1078-0432.CCR-20-1191 32605908

